# Effect and Mechanism of Survivin on Hypoxia-Induced Multidrug Resistance of Human Laryngeal Carcinoma Cells

**DOI:** 10.1155/2019/5696801

**Published:** 2019-04-24

**Authors:** Dan Xu, Da Wei Li, Jin Xie, Xin Wei Chen

**Affiliations:** ^1^Department of Otolaryngology-Head and Neck Surgery, Shanghai Jiao Tong University Affiliated Sixth People's Hospital, Shanghai 200233, China; ^2^Center for Translation Medicine, Yangpu Hospital, Tongji University School of Medicine, Shanghai 200031, China; ^3^Department of Otolaryngology-Head and Neck Surgery, Shanghai Jiaotong University Affiliated First People's Hospital, Shanghai 200080, China

## Abstract

This study aimed at clarifying the mechanism and role of survivin in hypoxia-induced multidrug resistance (MDR) of laryngeal carcinoma cells. Human laryngeal cancer cells were incubated under hypoxia or normoxia. The expression of* survivin* was silenced by performing RNA interference. Additionally, by Western blot and real-time quantitative RT-PCR,* survivin *expression was detected. The sensitivity of human laryngeal carcinoma cells to multiple drugs was measured by CCK-8 assay. Meanwhile, the apoptosis of cells induced by cisplatin or paclitaxel was assessed by Annexin-V/propidium iodide staining analysis. Under hypoxic conditions, the upregulation of survivin was abolished by RNA interference. Then, CCK-8 analysis demonstrated that the sensitivity to multiple agents of laryngeal carcinoma cells could be increased by inhibiting survivin expression (*P* < 0.05). Moreover, Annexin-V/propidium iodide staining analysis revealed that decreased expression of survivin could evidently increase the apoptosis rate of laryngeal carcinoma cells that were induced by cisplatin or paclitaxel evidently (*P* < 0.05). Our data suggests that hypoxia-elicited survivin may exert a pivotal role in regulating hypoxia-induced MDR of laryngeal cancer cells by preventing the apoptosis of cells induced by chemotherapeutic drug. Thus, blocking survivin expression in human laryngeal carcinoma cells may provide an avenue for gene therapy.

## 1. Introduction

Human laryngeal cancer has been considered as one of the most prevalent malignancies in the head and neck region. It has been known that chemotherapy is an important therapeutic method for the patients with advanced laryngeal carcinoma. Multidrug resistance (MDR), however, has still been a major obstacle to the effective management of chemotherapy in laryngeal carcinoma. Presently, the pathogenetic mechanisms regulating MDR in human laryngeal cancer are not well known.

It is well known that disequilibrium between the malignant cells' speedy proliferation and the irregular local vasculature can result in the formation of hypoxic areas within solid neoplasms. Accordingly, hypoxia has been regarded as a primary characteristic of human solid neoplasms including laryngeal cancer, which influences a series of cell biologic behaviors such as angiogenesis, stemness, invasion, and metastasis by both gene expression regulation and protein modification [1]. Moreover, it was confirmed that hypoxia could induce chemoresistance in a set of human malignant tumors [2–4]. Likewise, we have previously identified that hypoxia participates in regulating multidrug resistance in human laryngeal carcinoma [5]. Nevertheless, the regulatory mechanisms for MDR to chemotherapy in hypoxic laryngeal carcinoma cells are still not fully clarified.

Survivin, which is a member of the inhibitor of apoptosis protein (IAP) family, has been confirmed to be able to inhibit cell apoptosis through caspase-independent or caspase-dependent pathways [6]. As far as we know, survivin is usually overexpressed in the majority of malignancies [7–9]. Moreover, several literatures have illustrated that survivin overexpression is correlated with poorer prognosis in human laryngeal carcinoma [10, 11]. Similarly, our study has previously identified that overexpression of survivin is obviously associated with worse prognosis in laryngeal cancer [12]. In addition, survivin has been confirmed to be complicated in the regulation of MDR in various types of human tumour cells and silencing survivin could be seemed as an effective way to reverse multidrug resistance [13, 14]. Nevertheless, we have not found any reports about the role of survivin in regulating multidrug resistance of laryngeal carcinoma so far. On the other hand, our previous data have elucidated that hypoxia could induce multidrug resistance of laryngeal cancer cells [5] and promote the expression of survivin in laryngeal cancer cells [15]. However, whether survivin is involved in the regulation of MDR induced by hypoxia in laryngeal carcinoma is still not clear.

In the current study, we explored the effect of survivin on hypoxia-induced MDR of laryngeal cancer cells and its possible action mechanisms.

## 2. Materials and Methods

### 2.1. Cell Culture

Human laryngeal cancer cell line Hep-2 (ATCC® CCL-23™) was gained from the American Type Culture Collection (ATCC) about 10 years ago. Moreover, the AMC-HN-8 cell line was obtained from Asan Medical Center, Ulsan University College of Medicine about 8 years ago. We have checked that our laryngeal cancer cell lines have not been contaminated before the study. Both laryngeal cancer cell lines were grown in Dulbecco's modification of Eagle's medium (DMEM; Gibco Corporation, USA), supplemented with 10% fetal bovine serum (Hyclone, USA) and antibiotics (100 IU/ml streptomycin and 100 IU/ml penicillin) at 37°C in a humidified atmosphere with 5% CO_2_.

### 2.2. Exposure to Hypoxia

Human laryngeal carcinoma cells were cultured in a modulator incubator chamber (Nuaire™ US autoflow CO_2_ water jacketed incubator) at 37°C with 5% CO_2_ and 1% O_2_, balanced by N_2_.

### 2.3. Transfection of siRNA

The double-strand siRNA oligonucleotide that targets human survivin gene (sense: 5-GCAUUCGUCCGGUUGCGCUTT-3, anti-sense: 5-AGCGCAACCGGACGAAUGCTT-3) was synthesized by Shanghai Genepharma Co. Ltd. (China), which was reported previously [16]. Equally important, laryngeal cancer cells were transfected with a nonspecific control siRNA (sense: 5-UUCUCCGAACGUGUCACGUTT-3, antisense: 5–ACGUGACACGUUCGGAGAATT–3) as a negative control. The cells must be incubated in antibiotics-free medium for 24 hours prior to transfection with siRNA (100 nM) applying Lipofectamine 2000 (Invitrogen). These cells ought to be harvested and checked over after transfection for an additional 24 hours.

### 2.4. Real-Time Quantitative RT-PCR (qRT-PCR) Analysis

RNA was extracted from AMC-HN-8 and Hep-2 cells using Trizol reagent (Invitrogen) according to instructions of the manufacturer. As previously described [15], the isolated RNA was reverse-transcribed into complementary DNA (cDNA).* Survivin* primers were forward 5-CTTCATCCACTGCCCCAC-3; reverse 5-ACTTTCTCCGCAGTTTCCTC-3.* GAPDH* primers were forward 5-CATCTTCCAGGAGCGAGA-3; reverse 5-TGTTGTCATACTTCTCAT-3. PCR amplification was operated in a 20 *μ*L final reaction mixture including 10 *μ*M of each primer, 10 *μ*L SYBR Green PCR Master Mix, and a diluted cDNA solution. And then, the thermal cycling system was exhibited as the following: one cycle at 95°C for 10 minutes, 40 cycles at 95°C for 15 seconds, and at 60°C for 60 seconds. The analysis of the experimental data was assessed by the 2^−△△CT  ^ method as previously performed [17].

### 2.5. Western Blot Analysis

According to experimental requirement, laryngeal carcinoma cells were lysed with a cold radioimmune precipitation (RIPA) protein lysis buffer for 30 minutes. Equal quantities of protein extracts (25 *μ*g) were loaded onto SDS-PAGE (5% stacking gel and 15% separating gel), followed with a separation at 80 V for 2 hours and shifted onto PVDF membranes (Millipore), sealed with 4% skimmed milk for 1.5 hours at ambient temperature. Then, they were incubated with primary antibodies overnight at 4°C (Survivin 1:1000, rabbit anti-human; GAPDH, 1:1000, mouse anti-human) followed by HRP-conjugated anti-mouse or anti-rabbit secondary antibodies (1: 5000; ambient temperature, one hour). Ultimately, protein bands were visualized by electrogenerated chemiluminescence (ECL) in accordance with instructions of the manufacturer (Amersham Biosciences).

### 2.6. Analysis of Drug Resistance of Laryngeal Cancer Cells

CCK-8 assay was adopted to detect laryngeal cancer cells' sensitivity to adriamycin, 5-FU, paclitaxel, gemcitabine, and cisplatin. In brief, cancer cells were seeded in 96-well cell culture panels (5 × 10^3^ cells/well). After 12-hour incubation, drugs were added and then cultured for another 48 hours under hypoxic or normoxic conditions. The sensitivity of cells to different chemotherapeutic drugs was tested with CCK-8 assay. IC_50_ was measured as each drug's concentration required inducing a 50% reduction of cell population.

### 2.7. Annexin-V/Propidium Iodide Apoptosis Assay

The apoptotic indices (AI) of human laryngeal carcinoma cells were identified by flow cytometry analysis. Cancer cells in the period of logarithmic growth were plated in six-well panels (3 × 10^5^ cells/well, Hep-2; 4 × 10^5^ cells/well, AMC-HN-8) and incubated overnight at 37°C. Then, culture medium was resumed, and cell culture was continued under hypoxic or normoxic conditions for 12 hours. Cisplatin or paclitaxel was placed into each hole at a final concentration of 2.5 × 10^−9^ M or 5.0 × 10^−9^ M, respectively. After that, cancer cells were incubated in normoxia or hypoxia for further 48 hours. Next, 5 *μ*L (50 *μ*g/ml) of Annexin-V-FITC was added to cancer cells, and the cells were cultured for another 10 minutes. Finally, the cells were resuspended in 190 *μ*L of Tris-HCl buffer after being washed twice in DMEM. Meanwhile, 5 *μ*L (20 *μ*g/ml) of propidium iodide (PI) was added to cancer cells and incubated at 4°C for 10 minutes. The average fluorescence intensity of Annexin-V-FITC/PI was analyzed by flow cytometry, and the AI was estimated by the average fluorescence intensity.

### 2.8. Statistical Analysis

All experimentations were repeated no less than three times. Data were presented as mean ± standard deviation (SD). By Student's t-test or one-way ANOVA analysis, the quantitative variables were compared with SPSS13.0 statistical software (SPSS Inc., Chicago, Illinois, USA).* P* value of less than 0.05 was regarded as statistically significant.

## 3. Results

### 3.1. Inhibition of* Survivin* Gene Expression by RNA Interference

**Figure 1:**
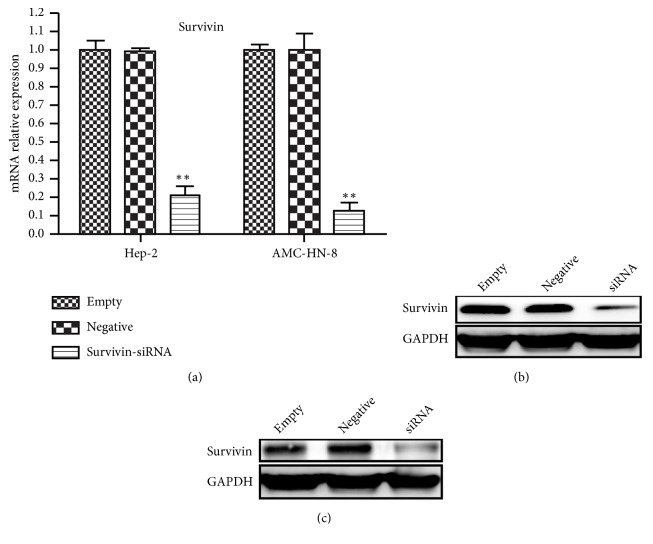
Downregulated* survivin* expression in hypoxic laryngeal carcinoma cells transfected with* survivin*-siRNA. AMC-HN-8 and Hep-2 cells were transfected with either a vector carrying a* survivin *targeting sequence (*survivin*-siRNA) or a vector carrying a* survivin* scrambled. Cells were cultured in hypoxia for 24 hours. By real-time quantitative RT-PCR,* survivin* mRNA expression in cells was detected; *∗∗P* < 0.01 vs. negative and empty groups in hypoxia (a). The expression of survivin protein in Hep-2 (b) and AMC-HN-8 (c) cell lines was measured by Western blot analysis.

Our work has previously shown that* survivin* expression in human laryngeal carcinoma cells could be obviously upregulated by hypoxia [15]. To explore the role of survivin in hypoxia-induced MDR in human laryngeal neoplasm, AMC-HN-8 and Hep-2 cells were firstly transfected with either a double-strand siRNA oligonucleotide that targeted* survivin* (s*urvivin*-siRNA) or scrambled siRNA for 24 hours before another 24-hour incubation in hypoxia. And then, the inhibitory effect was analyzed by Western blot and qRT-PCR. In comparison with the negative control or untreated control, both mRNA and protein expression of* survivin* gene in hypoxic laryngeal carcinoma cells were evidently downregulated after transfection of s*urvivin*-siRNA (Figure 1, *P* < 0.05). It suggested that siRNA of* survivin* could inhibit hypoxia-induced* survivin *expression.

### 3.2. Multidrug Resistance of Hypoxic Laryngeal Cancer Cells Was Suppressed by Inhibition of Survivin Expression

To explore whether survivin was involved in the MDR of laryngeal carcinoma cells induced by hypoxia, both AMC-HN-8 and Hep-2 cells had been transfected with scrambled siRNA or* survivin*-siRNA for 24 hours before being cultured in hypoxia. In these cells, the drug susceptibility of the* survivin*-siRNA group was compared to that of the control groups by applying CCK-8 assay. The CCK-8 assay data showed blocking survivin expression by transfected siRNA targeting* survivin* in hypoxic laryngeal carcinoma cells could strikingly elevate sensitivity to doxorubicin, gemcitabine, paclitaxel, cisplatin, and 5-FU under hypoxic conditions (Tables 1 and 2, *P* < 0.05). It demonstrated that knockdown of survivin in laryngeal cancer cells might partly reverse the hypoxia-induced MDR.

### 3.3. Survivin Preserves Hypoxic Laryngeal Carcinoma Cells from Apoptosis Induced by Cisplatin or Paclitaxel

**Figure 2:**
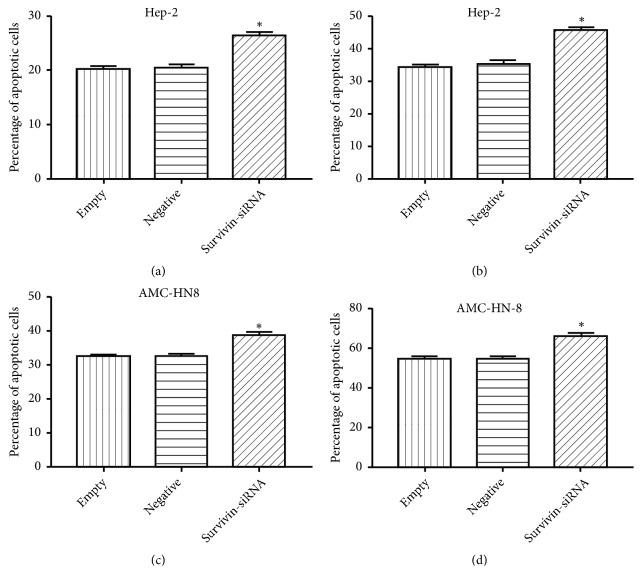
Effects of survivin expression on cell apoptosis induced by cisplatin or paclitaxel. The percentage of apoptotic laryngeal cancer cells in* survivin*-siRNA group and control groups induced by cisplatin or paclitaxel was analyzed by flow cytometry. *∗P* < 0.05: cells/*survivin*-siRNA versus cells/controls under hypoxia. (a) The percentage of apoptotic Hep-2 cells induced by cisplatin. (b) The percentage of apoptotic Hep-2 cells induced by paclitaxel. (c) The percentage of apoptotic AMC-HN-8 cells induced by cisplatin. (d) The percentage of apoptotic AMC-HN-8 cells induced by paclitaxel.

Our work has previously confirmed that hypoxia could decrease the apoptosis of laryngeal cancer cells that are induced by chemotherapeutic drug [5]. In our study, Annexin V/PI staining was used to evaluate the role of survivin in hypoxic protection of laryngeal carcinoma cells from apoptosis which was induced by cisplatin or paclitaxel. As can be seen from Figure 2, laryngeal carcinoma cells transfected with* survivin*-siRNA could evidently upregulate the rate of apoptosis induced by cisplatin or paclitaxel under hypoxia (*P* < 0.05). It could be seen from these data that survivin could play a role in preventing laryngeal carcinoma cells from chemotherapeutic drug-induced apoptosis under hypoxic conditions.

## 4. Discussion

To the best of our knowledge, multidrug resistance (MDR) has become a main therapeutic obstacle to the effectual chemotherapy of human laryngeal cancer. Consequently, the pathogenetic mechanisms involved in MDR of laryngeal cancer cells are worth further exploring. In human solid tumors, hypoxia, which has been recognized as a common feature, could subject tumor cells to a set of functional adaptive behaviors. According to the recent literature, hypoxia can contribute to the development of MDR in various human neoplasms, such as lung cancer [3] and hepatocellular carcinoma [4]. Meanwhile, our previous study has indicated that MDR of laryngeal cancer cells could be induced by hypoxia [5]. Accordingly, the underlying mechanisms of hypoxia-induced MDR in laryngeal carcinoma cells are worth further exploring. As a major member of the inhibitor of apoptosis family, survivin could be regarded as a key regulator of multiple biological behaviours in human cells, such as cell apoptosis, proliferation, and cell-cycle regulation [6]. Previously, our study of tissue samples has demonstrated that survivin expression in human laryngeal cancer tissues was obviously higher than that in laryngeal mucosal tissues, and its upregulation was correlated with advanced differentiation degree, stage, and lymphatic metastasis of laryngeal cancer [15], nearly in line with previous reports from other researchers [10, 11]. It was elucidated that survivin could be considered to be an effective biomarker for prognosis of human laryngeal cancer. Furthermore, several studies in the past few years have indicated that survivin might participate in the regulation of invasion and apoptosis of laryngeal carcinoma cells [18, 19]. Additionally, some authors have reported that survivin could play a significant role in the regulation of MDR in human colorectal [20] and breast cancer cells [14, 21], elucidating that inhibition of survivin expression might be an effectual approach to reverse MDR of human cancer. On the other hand, a set of researches have already illustrated that hypoxia could contribute to MDR of various human tumor cells [3, 4]. Consistent with these researches, our previous work has elucidated that hypoxia could induce MDR in laryngeal cancer cells [5]. Equally important, our data have previously clarified that hypoxia could promote survivin expression in laryngeal cancer cells [15]. To further determine whether survivin contributed to the regulation of MDR induced by hypoxia in laryngeal carcinoma cells, we investigated the drug susceptibility to cisplatin, 5-FU, paclitaxel, gemcitabine, and adriamycin in treating human laryngeal cancer cells. Consequently, the current research confirmed that suppression of survivin expression could evidently increase the chemosensitivity of laryngeal carcinoma cells to multiple drugs in hypoxic environments, which further demonstrated that survivin might partly take part in MDR induced by hypoxia in laryngeal carcinoma. That is to say, suppression of survivin expression could be considered as an effective manner to resist multidrug resistance of laryngeal cancer.

Theoretically, protection from induced apoptosis could be regarded as a crucial mechanism of multidrug resistance in tumour cells. Until now, it has been proven that suppression of survivin expression could obviously induce the apoptosis of laryngeal carcinoma cells in multiple in vitro studies [18, 22]. Previously, our study has also confirmed that upregulation of survivin expression was significantly associated with decreased apoptosis index in human laryngeal squamous cell carcinoma tissues [12], further sustaining the notion that survivin is involved in the regulation of laryngeal cancer cells apoptosis. Furthermore, several literatures have shown that survivin plays a key role in regulating drug resistance by its biologic function of resistance to apoptosis in multiple types of neoplasias [23, 24]. In this series, our work demonstrated that blocking survivin expression could significantly promote the apoptosis induced by cisplatin or paclitaxel in hypoxic laryngeal carcinoma cells, reversing drug resistance. As stated above, our data demonstrated that survivin might contribute to hypoxia-induced multidrug resistance in laryngeal carcinoma cells via regulating the function for apoptosis inhibition. In vivo study on the mechanism and role of survivin in hypoxia-induced MDR of laryngeal cancer cells will be performed by using mice transplant tumor model in our further research. Synchronously, the molecular mechanisms involved in regulating survivin expression in hypoxic laryngeal cancer cells will also be investigated.

## 5. Conclusions

In summary, our study indicates that survivin may play an important role in regulating MDR in laryngeal carcinoma cells under hypoxia. Equally important, according to our data, the possible mechanism for survivin that contributes to MDR is the resistance to cell apoptosis induced by drug. Consequently, targeting the survivin signal pathway may be a potential strategy for reversing MDR of human laryngeal cancer.

## Figures and Tables

**Table 1:** Effect of survivin expression inhibition on chemosensitivity in hypoxic Hep-2 cells.

Drug	IC_50_ (*μ*g/ml)		
	Untreated control	Negative control	Survivin-siRNA
Paclitaxel	39.05×10-3±0.22	39.43×10-3±0.18	11.42×10-3±0.39*∗*
5-Fu	244.23±0.32	242.63±0.58	93.38±0.53*∗*
Doxorubicin	3.93±0.13	3.88±0.15	2.85±0.37*∗*
Gemcitabine	37.90±0.28	38.49±0.18	29.58±0.43*∗*
Cisplatin	8.95±0.15	9.14±0.14	5.82±0.30*∗*

IC_50_ is the concentration of each drug that caused a 50% reduction in the number of cells.

Mean ± SD of three individual experiments are shown. *∗*: *P* < 0.05 vs. Untreated control and Negative control.

**Table 2:** Effect of survivin expression inhibition on chemosensitivity in hypoxic AMC-HN-8 cells

Drug	IC_50_ (*μ*g/ml)		
	Untreated control	Negative control	Survivin-siRNA
Paclitaxel	37.22×10-3±0.14	37.31×10-3±0.15	10.62×10-3±0.32*∗*
5-Fu	234.28±0.27	227.12±0.18	92.22±0.41*∗*
Doxorubicin	3.84±0.83	3.80±0.28	2.67±0.14*∗*
Gemcitabine	36.77±0.12	37.24±0.19	27.62±0.18*∗*
Cisplatin	8.22±0.36	8.52±0.42	5.72±0.14*∗*

IC_50_ is the concentration of each drug that caused a 50% reduction in the number of cells.

Mean ± SD of three individual experiments are shown. *∗*: *P* < 0.05 vs. Untreated control and Negative control.

## Data Availability

The datasets used and/or analysed during the current study are available from the corresponding author on reasonable request.
